# Ethical, legal, and policy dimensions and contentions for reanalysis and reinterpretation of clinical genetic testing results

**DOI:** 10.3389/fgene.2025.1685854

**Published:** 2025-10-08

**Authors:** Zachary T. Sentell, Ma’n H. Zawati

**Affiliations:** ^1^ Department of Human Genetics, McGill University, Montreal, QC, Canada; ^2^ Centre of Genomics and Policy, Victor Phillip Dahdaleh Institute of Genomic Medicine, McGill University, Montreal, QC, Canada

**Keywords:** clinical genetic testing, medical genetics, variant classification, reanalysis, variant reinterpretation, ethical considerations, legal, policy

## Abstract

The rapid evolution of genomic knowledge has made reanalysis and reinterpretation of clinical genetic testing results an ethical imperative to ensure optimal patient care. However, significant discrepancies persist between policies, laboratory practices, and stakeholder perspectives regarding the responsibility for initiating and communicating reclassified variants. This perspective examines the current landscape of ethical, legal, and practical challenges for laboratories, clinicians, and patients. We highlight the tension between the duty of care and resource constraints, finding that while the ethical importance of reinterpretation is acknowledged, the lack of standardized guidelines and legal clarity fuels uncertainty and discordant stakeholder views. To address these challenges, we propose an actionable, shared-responsibility framework that aligns duties with expertise. In this model, diagnostic laboratories are positioned to monitor new evidence and initiate updates for reinterpretation, while clinicians manage patient recontact and initiate case-level reanalysis, and health systems provide the necessary infrastructure. Realizing this framework through multidisciplinary collaboration and investment is crucial for establishing equitable best practices and integrating reinterpretation into the evolving standard of care.

## 1 Introduction

Clinical genetic testing has revolutionized diagnostic capabilities for genetic disorders, offering diagnostic yields of 25%–40% in rare conditions (Wojcik et al., 2023). However, the dynamic nature of genomic knowledge necessitates periodic reinterpretation of genetic data to ensure clinical relevance. Systematic reanalysis can provide diagnoses for an additional 13%–22% of previously unsolved cases, underscoring its potential to improve patient care (Hartley et al., 2023; Tan et al., 2020; Machini et al., 2019). Variant classification, governed by guidelines from the American College of Medical Genetics and Genomics (ACMG) and the Association for Molecular Pathology (AMP), as well as ClinGen specifications, remains inherently fluid, with classifications evolving as new evidence emerges (Rehm et al., 2015; Harrison et al., 2019; Richards et al., 2015). Because diagnostic laboratories maintain the infrastructure for genomic data management, gene and variant curation, and evidence synthesis, they are best equipped to conduct ongoing variant surveillance, whereas clinicians remain responsible for ensuring that updated results are communicated to patients through recontact when clinically indicated.

The implications of variant reinterpretation are profound. Reclassification can alter clinical management strategies, including treatment plans, surveillance protocols, and preventive interventions such as cancer risk-reducing surgeries (Murray et al., 2011; El Mecky et al., 2019; Walsh et al., 2024). A recent scoping review highlighted the fluid nature of variant classification, reporting that proactive reinterpretation changed the classifications for an average of 31% of variants (ranging from 4.7% to 100%), while routine clinical practice led to reclassification in approximately 20% of cases, predominantly affecting variants of uncertain significance (VUS) (Thummala et al., 2024). For patients receiving genetic testing for hereditary cancer syndromes, 7% had reclassified variants and of those reclassifications about 12% had the potential to significantly change clinical care (Turner et al., 2019). Despite these benefits, the field lacks consensus on the optimal frequency and mechanisms for initiating reinterpretation. Reported reclassification rates vary (3.6%–58.8%), with most occurring within 2 years of initial reporting (Walsh et al., 2024; Wright et al., 2018). Longitudinal analysis of hereditary cancer genetic testing (n > 1.9 million) revealed a 4.7% variant reclassification rate over two decades (Esterling et al., 2020). Notably, while most were downgrades, approximately 20% of these reclassifications represented upgrades to pathogenic or likely pathogenic status. The clinical significance of variant reinterpretation is demonstrated by a study in arrhythmogenic right ventricular cardiomyopathy, where over 40% of reclassified variants had implications for patient management and 10% of patients had a variant downgrade that led to a loss of their disease status (Costa et al., 2021). This emphasizes the importance of an accurate genetic diagnosis and periodic reassessment of variant pathogenicity classifications.

Responsibility for initiating reinterpretation remains a contentious issue. Laboratories are generally not obligated to perform reinterpretation unless prompted by clinicians or patients (Clayton et al., 2021). This reactive approach contrasts with calls for systematic frameworks that integrate reinterpretation into routine practice. However, significant barriers persist, including resource constraints, inconsistent policies, and unclear legal obligations. These challenges are exacerbated by discordant stakeholder perspectives on who bears responsibility for initiating and communicating reclassified results.

Clinical genetic testing presents a persistent ethical challenge: as variant interpretation evolves, who is responsible for ensuring patients receive updated information that could impact their care? Despite the clear benefits of reanalysis and reinterpretation, significant gaps remain, including inconsistent guidelines, unclear responsibilities, discordant stakeholder perspectives, and systemic barriers (Chisholm et al., 2018; Appelbaum et al., 2023). This perspective critically examines these ethical, legal, and policy dimensions, highlighting the need for standardized frameworks to align ethical imperatives with practical constraints and proposing actionable recommendations to ensure equitable and effective practices in genomic medicine.

## 2 Legal considerations

Currently, no laws explicitly mandate the routine reinterpretation of clinical genetic test results, and courts have yet to impose liability for failing to reanalyze or recontact patients with updated genetic findings (Clayton et al., 2021). While ethical imperatives to preserve and reassess genetic data are widely acknowledged, legal frameworks remain ambiguous, leaving laboratories and clinicians uncertain about their obligations. For example, the ACMG recommends that laboratories establish protocols for variant re-evaluation and case-level reanalysis (Deignan et al., 2019), but practices vary across institutions (El Mecky et al., 2019). In Canada, the Canadian College of Medical Geneticists (CCMG) similarly proposes reinterpretation as a shared responsibility among healthcare professionals rather than a legal duty (Goh et al., 2024).

The courts have not imposed a legal obligation, as highlighted by *Williams v. Quest/Athena*, where a laboratory’s initial classification of a variant later reclassified as pathogenic was central to litigation following a child’s death (GenomeWeb, 2016). The plaintiff argued that if the variant had been correctly classified initially, alternative treatment could have been pursued, potentially preventing the child’s death. The court ruled in favor of the laboratory, citing insufficient evidence to establish causation between the misclassification and patient outcomes (GenomeWeb, 2020). This case highlights the unresolved legal questions surrounding liability for variant misclassification or delayed reinterpretation (McGrath et al., 2021).

Laboratory obligations for reinterpretation requests often depend on regional data protection laws. Laboratories are generally required to provide existing reports or raw data upon patient request but are not obligated to perform reinterpretation unless prompted by clinicians (Clayton et al., 2021). Reinterpretation is typically reactive, initiated by clinicians in response to new clinical information or evolving patient phenotypes (Clayton et al., 2021). While laboratories and physicians do not have a legal obligation to routinely reassess genetic test results, this is an emerging area of focus within the field.

Marchant *et al.* underscore the importance of revisiting legal liabilities in the context of clinical genetics to ensure that guidelines evolve with technological advances (Marchant et al., 2020). The concept of treating genomic data as a *lifetime resource* raises concerns about future liability for unreported findings or secondary variants (Marchant et al., 2020). To mitigate these risks, many laboratories currently favor targeted testing approaches limited to well-characterized genes immediately relevant to diagnosis. Conversely, Roberts and Foulkes argue that clinically meaningful variant reclassifications *should* carry a legal duty for the laboratory and the ordering clinician, presenting a potential framework for liability (Roberts and Foulkes, 2020). Unsurprisingly, there is a high degree of concern for liability of reinterpretation and recontact among both clinical genetic and laboratory genetic providers (Berger et al., 2022).

The existing consensus holds that laboratories are not legally compelled to undertake reanalysis or reinterpretation of clinical genetic tests (Vears et al., 2018; Matthijs et al., 2016; Geest et al., 2024). Although routine reanalysis is not the standard of care, practices may evolve to establish reinterpretation as a legal obligation (Clayton et al., 2021; Marchant et al., 2020; Foulkes et al., 2020). This lack of clarity creates uncertainty for laboratories and clinicians.

## 3 Ethical imperatives

The ethical debate surrounding the reinterpretation of genetic test results centers on the responsibilities of laboratories, clinicians, and healthcare systems in ensuring patients benefit from updated knowledge and new technology. Beneficence, the moral obligation to act in the patient’s best interest, is frequently invoked to argue for reanalysis and reinterpretation to improve clinical care. For example, reclassification of variants can lead to actionable changes in treatment plans or surveillance strategies, underscoring the potential for significant patient benefit (Supplementary Table S1) (Appelbaum et al., 2020). Non-maleficence, the duty to avoid harm, is critical, as failing to reinterpret variants or communicate updated results may prevent patients from having opportunities for improved care or expose them to unnecessary interventions based on inaccurate information.

While there is no recognized legal duty to take action on reclassified genetic test results, there is an ongoing debate regarding the extent of the responsibilities borne by the laboratories, clinicians, healthcare system, and patients (Roberts and Foulkes, 2020).

Appelbaum *et al.* argue that ordering genetic tests creates an ethical imperative to continually reinterpret variants as knowledge evolves, obligating providers to update and communicate new findings (Appelbaum et al., 2020). Although logistical and resource constraints exist, they maintain that ethical duties outweigh these barriers and predict that advancing technologies will soon make reinterpretation a standard of care (Appelbaum et al., 2020). Furthermore, they assert that laboratories carry an ongoing duty (beyond legal requirements) to proactively reanalyze results, warning that failure to do so risks abandoning patients (Appelbaum et al., 2020).

Watts and Newson contend that universal, systematic reinterpretation is impractical given current logistical and financial constraints and instead endorse reactive, clinician-triggered reinterpretation (Watts and Newson, 2023). They argue that although clinicians have a continuing duty of care to patients that are undergoing clinical genetic testing, this does not extend to routine reassessment of all VUSs (Watts and Newson, 2023). They argue that despite offering genetic tests that have the potential to yield clinically meaningful results in the future if reliably reinterpreted, these genetic tests are not currently offered with this condition (Watts and Newson, 2023). Instead, laboratories should focus on reevaluating pathogenic variants, especially those with a high risk of false positives or those common in underrepresented populations, while making two exceptions: VUSs identified as potentially clinically relevant and cases where laboratories receive explicit payment for reinterpretation services (Watts and Newson, 2023).

Equity is a particularly critical issue in the context of variant reinterpretation. Individuals from minority populations are disproportionately affected by VUSs due to limited representation in genomic databases (Walsh et al., 2024; Plon and Rehm, 2018; Slavin et al., 2018; Makhnoon et al., 2023; Caswell-Jin et al., 2018). These disparities result in fewer definitive diagnoses, a higher burden of VUSs, and higher rates of genetic misclassification, exacerbating health inequities (Manrai et al., 2016). Slavin *et al.* demonstrated that rates of variant re-classification differ significantly across ancestries; for example, 18% of *BRCA1/2* variants were reclassified over 20 years, with minority populations benefiting most from these updates (Slavin et al., 2018). This highlights the essential role of VUS reinterpretation in upholding effective clinical care (Popejoy and Fullerton, 2016). Addressing these inequities requires expanding genomic datasets to include diverse populations and integrating routine reinterpretation into clinical practice (Veenstra et al., 2021). Doing so would improve diagnostic accuracy and reduce disparities in access to actionable genetic information (Lee et al., 2022).

Despite the significant practical limitations, there is mounting pressure for laboratories to assume the responsibility of *actively* or *proactively* reinterpreting variants, and that this ethical responsibility should inform policymaking and the development of best practices (Watts and Newson, 2023; Appelbaum et al., 2020). The ethical debate surrounding the reinterpretation of genetic test results highlights the tension between idealistic commitments to beneficence, non-maleficence, autonomy, and equity, and the practical realities faced by laboratories and clinicians.

## 4 Policy gaps

Reanalysis and reinterpretation of clinical genetic test results are guided by recommendations from professional societies, including the American College of Medical Genetics and Genomics (ACMG), the Canadian College of Medical Geneticists (CCMG), and the Association for Clinical Genomic Science (ACGS). While the organizations emphasize an ethical importance, there remains a lack of clarity and discordance on the responsibilities for reinterpretation. The key guidelines and position statements are summarized in Table 1.

**TABLE 1:** Statements for the reanalysis and reinterpretation of genetic test results.

Organization	Statements
American College of Medical Genetics and Genomics (ACMG) Laboratory Quality Assurance Committee (Deignan et al., 2019)	• The ACMG recommends that clinical laboratories establish policies and protocols for variant re-evaluation and case-level reanalysis and respond to requests for these services in a timely manner. If laboratories perform active variant reinterpretation, there should be policies in place for clinically significant variant reclassifications including the communication of an updated report to the ordering provider • Clinical laboratories should report and communicate reclassified variants that could impact patient management
American College of Medical Genetics and Genomics (ACMG) Social Ethical Legal Issues Committee (David et al., 2019b)	• The ACMG considers recontact as a fundamental shared responsibility involving the ordering healthcare provider, the clinical testing laboratory, and the patient • Patients should be informed pre-test that interpretations can change. They should be advised to update providers about clinically-relevant changes and that they can initiate requests for an update on an uncertain result by contacting the provider. It is the patient’s responsibility to update the provider with relevant medical and contact information • Upon receiving updated information from the lab, the ordering physician should make reasonable efforts to recontact the patient • The clinical laboratory holds the responsibility to inform the ordering physician of significant variant reclassifications or the discovery of new gene-disease relationships
Canadian College of Medical Geneticists (CCMG) (Goh et al., 2024)	• The CCMG frames recontact as a shared responsibility involving patients, ordering clinicians, and laboratories • Recontact in the Canadian public healthcare system is not a recognized duty • Clinicians are advised to discuss potential interpretation changes and recontact pathways with patients • Laboratories should establish clear policies for handling reinterpretation requests but have no current duty to systematically search for updated variant information or other patients with the same variant • Practitioners must ensure patient agreement and adherence to laboratory policy when requesting reinterpretation, while laboratories should establish and communicate clear policies and processes for such requests
The Association for Clinical Genomic Science (ACGS) (Durkie et al., 2024)	• The ACGS recommends that laboratories must store variant data and relevant information to facilitate future reclassification • Reclassifications with clinical relevance should be shared with healthcare professionals, prioritizing the communication of changes that significantly impact clinical management
European Society of Human Genetics (ESHG) (Matthijs et al., 2016)	• The ESHG states that the laboratory does not have the responsibility to systematically reanalyze previous data and report reclassified findings • However, when the laboratory’s classification of a variant changes (e.g., VUS to Pathogenic), the lab must reanalyze data, update reports, recontact clinicians of potentially affected patients, necessitating a system to effectively link patients and variants for retrieval upon reclassification
American Society of Human Genetics (ASHG) (Bombard et al., 2019)	• In a research context, the ASHG recommends attempting recontact if reinterpreted results could affect medical management or relate to the phenotype under study. Recontact is also advised for significant classification changes (to/from pathogenic/likely pathogenic) even if management is unaffected

In the United States, the ACMG Laboratory Quality Assurance Committee recommends that laboratories establish protocols for variant re-evaluation and case-level reanalysis but does not mandate routine reinterpretation or specify whether laboratories or clinicians should initiate the process (Deignan et al., 2019). However, a statement from the ACMG Social, Ethical, and Legal Issues (SELI) Committee suggests the laboratory bears responsibility for informing the ordering physician of significant variant reclassifications or newly established gene-disease relationships (David et al., 2019b), a position potentially incongruent with other guidance from the ACMG. Overall, the ACMG framework leans towards a shared responsibility model involving the laboratory, provider, and patient, noting that recontact often depends on patient initiative (David et al., 2019a). Similarly, Canadian guidance from the CCMG affirms the ethical responsibility and highlights a shared responsibility model among laboratories, clinicians, and patients but acknowledges that resource constraints and the absence of established protocols (Goh et al., 2024). The CCMG advisory document outlines a *push and pu*ll model for genetic result reinterpretation. Practitioners *pull* updates by requesting them based on time or clinical changes, while labs *push* significant reclassifications, prompting reasonable practitioner efforts to recontact patients (Goh et al., 2024). Patients should be made aware of reinterpretation opportunities and are advised to initiate contact if re-evaluation becomes relevant. Ordering clinicians are advised to proactively request variant reinterpretation from laboratories when clinical indications arise or when sufficient time has elapsed to warrant reanalysis. Diagnostic laboratories are recommended to develop specific policies for handling reinterpretation requests, including criteria for unsolicited reclassification updates. In cases where laboratories report clinically significant changes, clinicians are expected to make reasonable efforts to recontact the patient.

In the United Kingdom, the ACGS emphasizes the importance of storing variant data to facilitate future reclassification and advises laboratories to prioritize clinically significant updates (Durkie et al., 2024). Notably, reclassifications that affect clinical relevance should be shared with relevant healthcare professionals, and for variants impacting clinical management decisions, rapid dissemination of this information is advised (e.g., CanVar-UK database for cancer-susceptibility gene variants). However, like the ACMG and CCMG, it does not explicitly mandate proactive reinterpretation.

In contrast, research-focused organizations such as the European Society of Human Genetics (ESHG) and the American Society of Human Genetics (ASHG) adopt a more general stance on the ethical responsibility. While ESHG guidelines state laboratories are not obligated to proactively reinterpret results based on new evidence, they do mandate that if a lab reclassifies a variant, it must reanalyze, report, and recontact clinicians. This creates a significant ethical duty for reactive reinterpretation prompted by external triggers like clinician requests (Table 1) (Matthijs et al., 2016). The ASHG focuses on research contexts, recommending recontact when reinterpretation yields findings with clinical relevance (Bombard et al., 2019).

A notable gap in existing policies is the absence of clear guidance on defining the scope of reanalysis. While the shared ethical responsibilities for patients, ordering clinicians, and laboratories are broadly acknowledged, effective implementation requires standardized guidelines and improved infrastructure and resources (Goh et al., 2024).

## 5 Stakeholder discordance

There is discordance among laboratory providers, clinicians, and patients regarding roles, responsibilities, and expectations of reinterpretation and reanalysis in clinical genetic testing. While diagnostic laboratory directors largely view the responsibility of initiating reinterpretation as belonging to the ordering clinician and the patient (El Mecky et al., 2019), this view is not always shared; one study found that while 59% of clinicians believe labs should trigger reinterpretation, only 39% of lab directors agreed (Berger et al., 2022).

Most patients and clinical or laboratory providers were in favor of reporting *all new findings*, while the non-genetic providers prioritized *new results that could change the patient’s management*. Within the laboratory providers, the laboratory directors were less inclined to report a new result compared to laboratory genetic counselors (Berger et al., 2022). Most respondents across groups, including 74% of laboratory providers, believe there should not be a fixed end-point for the laboratory’s responsibility to reinterpret previously reported genetic variants (Berger et al., 2022). However, this raises questions about the systems and practices that will need to be implemented to allow for the long-term storage of clinical genomic data should this become the expected standard.

There are differing views on the consenting process in reinterpretation and recontact. While most genetics professionals did not feel specific re-consent was necessary prior to reinterpretation, patients and non-genetic providers favored explicit consent before reinterpretation occurs (Berger et al., 2022). Patient understanding of the reclassification process itself is often limited, however, most patients value being recontacted despite the associated challenges and generally view healthcare providers as responsible for initiating recontact (Otten et al., 2014). Genetic counselors express conflicting views on their role, with some accepting primary responsibility (Scherr et al., 2015) and others viewing it as shared or patient-led (Mueller et al., 2019). The validity and durability of initial consent obtained years prior, often without detailed discussion of future reanalysis possibilities, presents a significant challenge, particularly given that current practices sometimes involve reinterpretation without updated patient agreement (El Mecky et al., 2019; Geest et al., 2024). This highlights a critical need for a dynamic consent process and improved patient education regarding the evolving nature of genomic knowledge (El Mecky et al., 2019; David et al., 2019b; Appelbaum et al., 2023; Deignan et al., 2019).

Recontact is viewed as a shared duty, where clinicians handle results for their active patients, but healthcare system-level solutions are needed to ensure variant updates reach all affected individuals (David et al., 2019b; Goh et al., 2024). Clayton *et al.* suggest that laboratories should be cautious about returning reinterpreted results directly to patients, as this establishes a clinical relationship that carries potentially unforeseen responsibilities (Clayton et al., 2021). Marchant *et al.* further emphasize that laboratories do not necessarily have the means to recontact patients with reports of variant reclassifications. Rather, they suggest that if the practice of active variant reinterpretation emerges, then the responsibility may lie with the laboratory to contact the ordering physician with the reinterpreted results, where the responsibility would then remain with them to communicate the results to the patient (Marchant et al., 2020).

## 6 Actionable recommendations for a shared responsibility framework

The stakeholder discordance, conflicting expectations, and operational gaps described above demonstrate that while a shared responsibility framework is broadly endorsed in principle, it remains to be realized. To operationalize a shared responsibility model, roles must align with expertise. Our proposed framework clarifies this by distinguishing between proactive, variant-level reinterpretation initiated by diagnostic laboratories and reactive, case-level reanalysis initiated by clinicians in response to new patient information. Table 2 outlines these responsibilities, designed to support the integration of reinterpretation as a standard of care without asserting a new legal duty.

**TABLE 2:** Actionable recommendations for a shared-responsibility model.

Stakeholder group	Actionable recommendation
Diagnostic Laboratories	Maintain policy-based, criteria-triggered surveillance of previously reported patient variants (e.g., ACMG/AMP or ClinGen VCEP aligned updates)
	When criteria indicate a clinically significant change, issue an updated report to the ordering clinician
	Share reclassifications to public databases (e.g., ClinVar)
Ordering Clinicians	Integrate discussion of potential reinterpretation and recontact preferences into the pre-test consent process
	Timely initiation of case-level reanalysis or reinterpretation with the laboratory in response to significant changes in a patient’s clinical presentation or family history
	Upon receipt of an updated laboratory report, assess relevance to care and make reasonable efforts to recontact, per local policy
Patients	Keep contact information and relevant family history updated with their provider
	Patients may inquire about results if their personal or family health status changes
Health Systems and IT	Develop and maintain robust, interoperable infrastructure (e.g., EHR integration) that links patient records to genomic results over time
	Support dynamic consent and patient-initiated requests
	Implement validated, governed AI-augmented automation for variant surveillance and updates
Researchers, Funders and Policymakers	Develop and maintain living, harmonized guidelines that clarify roles and processes
	Fund initiatives that diversify genomic reference databases and establish clear reimbursement models for reinterpretation

## 7 Conclusion

Discordance persists over who is responsible for initiating reinterpretation and recontact. Combined with legal ambiguity, lack of harmonized policies, and equity gaps, these challenges limit the potential of genomic medicine. While a shared responsibility framework is broadly endorsed, operational and role-designation gaps remain. The actionable framework proposed here aims to resolve these issues by aligning duties with expertise. Bridging these gaps through collaboration and infrastructure innovation is essential to making reanalysis and reinterpretation an equitable standard of care.

## Data Availability

The original contributions presented in the study are included in the article/Supplementary Material, further inquiries can be directed to the corresponding author.
