# Supplementary material for: Ethical, legal, and policy dimensions and contentions for reanalysis and reinterpretation of clinical genetic testing results
Source: Front Genet. 2025 Oct 8;16:1685854. doi: 10.3389/fgene.2025.1685854 (PMC12542829; doi:10.3389/fgene.2025.1685854)
Supplement: Supplementary file 1 [file Table1.docx]

**Table 1. Ethical Principles to Consider for Reanalysis and Recontact**

| **Principle** | **Key Considerations** |
| --- | --- |
| Beneficence | - Improve patient care by communicating significant updates in genetic information. - Minimize psychological impact by providing clear, actionable health guidance with recontact. |
| Non-maleficence | - Avoid overburdening patients with unnecessary medical follow-up through careful reclassification. - Prevent psychological harm by considering the implications of recontact on patient well-being. |
| Autonomy | - Uphold patient choice regarding recontact, respecting individual preferences and consent. - Ensure clarity regarding consent, especially in cases where patient capacity to consent evolves over time. |
| Equity | - Ensure equitable recontact procedures that account for varying patient awareness and resource levels. - Continuously adapt recontact methods to reflect emerging evidence and maintain fairness. |
